# Embigin, regulated by HOXC8, plays a suppressive role in breast tumorigenesis

**DOI:** 10.18632/oncotarget.4360

**Published:** 2015-06-08

**Authors:** Fengmei Chao, Jun Zhang, Yang Zhang, Houli Liu, Chenchen Yang, Juan Wang, Yanjun Guo, Xiaohong Wen, Kaiye Zhang, Bei Huang, Daihai Liu, Yong Li

**Affiliations:** ^1^ Anhui University, School of Life Sciences, Center for Stem Cell and Translational Medicine, Hefei, Anhui Province, P. R. China; ^2^ Anhui University, School of Life Sciences, Hefei, Anhui Province, P. R. China; ^3^ The Key Laboratory of Developmental Genes and Human Disease, Ministry of Education, Institute of Life Science, Southeast University, Nanjing, P. R. China

**Keywords:** EMB, transcription, HOXC8, breast cancer, tumorigenesis

## Abstract

The transmembrane glycoprotein embigin (EMB) belongs to the immunoglobulin superfamily (IgSF) and a number of IgSF members have been identified as biomarkers for cancer progression. In this study, we show that embigin is transcriptionally regulated by Homeobox C8 (HOXC8) in breast cancer cells and embigin expression suppresses breast tumorigenesis. With aid of Western blot, luciferase reporter gene assay and chromatin immunoprecipitation, we reveal that HOXC8 binds to the EMB promoter at the region of nucleotides −2303 to −2315 and acts as a transcription inhibitor to suppress embigin expression. Depletion of embigin leads to increase in proliferation, anchorage-independent growth and migration of breast cancer cells, and the inhibitory effects mediated by HOXC8 knockdown on breast tumorigenesis can be largely rescued by depletion of embigin expression in breast cancer cells, suggesting that HOXC8 regulates breast tumorigenesis, at least partly, through regulating embigin expression. Moreover, we show that loss of embigin promotes proliferation, anchorage-independent growth, and migration ability of normal mammary epithelial MCF10A cells. The analyses of publically available human breast tumor microarray gene expression database show that low embigin levels correlate with short survival of breast tumor patients, particularly with basal-like tumor patients, and embigin expression is low specifically in patients with basal-like, ER-/HER2- tumors. Taken together, our study demonstrates that low/loss of embigin plays an important role in the progression of breast tumors.

## INTRODUCTION

Members of the immunoglobulin superfamily include a large number of cell adhesion molecules that mediate not only cell–cell or cell–matrix interaction but also many aspects of cell behavior, such as cell motility, proliferation, and differentiation, etc [1]. Embigin (EMB) is a transmembrane glycoprotein belonging to the immunoglobulin superfamily, and originally identified to express during mouse embryogenesis [2-4]. During mouse embryogenesis, embigin is strongly expressed in the endoderm in early postimplantation, and in the gut and visceral endoderm in the somite stage [4, 5]. Embigin is also detected in a variety of tissues including heart, liver, lung and brain, and involved in prostate and mammary gland development [3, 5]. It has been reported that embigin protein is expressed in a variety of prostate and mammary cancer cell lines, and its expression appears to be down-regulated in cancer cells upon Matrigel culture [4]. These data suggest that embigin plays an important role not only in embryo and tissue development but also probably in cancer development. However, very little is known about the regulation of embigin expression and its roles in cancer development.

HOXC8 belongs to homeobox (HOX) family consisting of 39 HOX genes, and has been reported to participate in a number of physiological and pathological processes including mouse embryogenesis and human tumorigenesis [6, 7]. HOXC8 is expressed in the neural tube and somatic mesoderm as well as in the prospective thorax during mouse embryogenesis, and also expressed in a variety of organs including brain, breast, placenta, liver, bone and nervous system [8, 9]. Previously we reported that HOXC8 plays an important role in promoting breast cancer development, correlating with other reports that HOXC8 expression is deregulated in several cancers, such as cervical cancer, prostate cancer, esophageal cancer, and pancreatic cancer, etc [10–14]. Although HOXC8 is important in tumorigenesis, its underlying mechanisms remain largely unclear. In mouse embryonic fibroblasts, HOXC8 was reported to regulate a number of genes expression including embigin [7, 15]. Therefore, it is our interest to investigate whether HOXC8 could function as the embigin transcription factor in breast cancer cells.

In this study, we show that HOXC8 binds to the embigin promoter and transcriptionally inhibits embigin expression, and loss of embigin expression enhances proliferation, anchorage-independent cell growth, and migration of breast cancer cells and normal breast cell MCF10A. HOXC8 knockdown mediated inhibition on breast cancer cell growth and migration can be reversed largely by removal of embigin protein expression, indicating the functional linking between embigin and HOXC8 in breast tumorigenesis. Using web-based survival analysis tools with publically available breast cancer microarray databases [16–18], we found that embigin expression is significantly low in patients with basal-like, ER-/HER2- tumor, the highest risk subtype of breast tumors, and Kaplan-Meier analysis shows that low expression of embigin correlate with the shorter survival of breast cancer patients, particularly with basal-like cancer patients. Taken together, our results showed that embigin is transcriptionally regulated by HOXC8 protein, and plays a suppressive role in breast cancer progression.

## RESULTS

### HOXC8 regulates embigin expression by inhibiting embigin transcription

**Figure 1:**
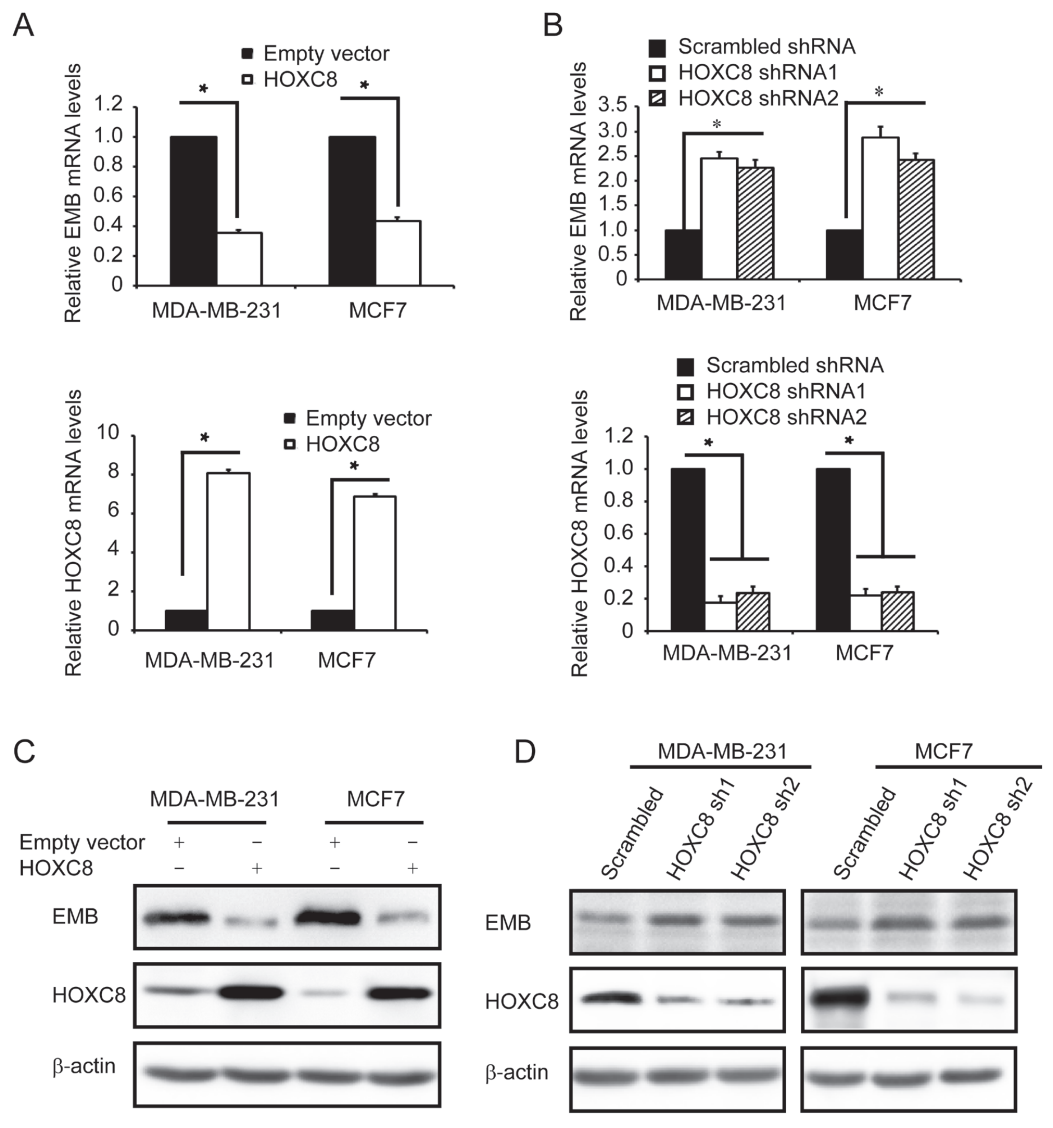
HOXC8 regulates embigin expression in MDA-MB-231 and MCF7 cell lines **A.** MDA-MB-231 and MCF7 cells were lentivirally transduced with empty vector or vector encoding HOXC8, and total RNA from these cells were subjected to qRT-PCR to determine the level of EMB mRNA (upper panel) and the level of HOXC8 mRNA (lower panel). β-actin and GAPDH mRNA were used as internal controls for standardization. Data are mean ± SE. *n* = 4. *P < 0.05. **B.** MDA-MB-231 and MCF7 cells were lentivirally transduced with vector encoding scramble sequence, or HOXC8 shRNAs. Total RNA was isolated from these cells and subjected to qRT-PCR to determine the level of EMB mRNA (upper panel) and the level of HOXC8 mRNA (lower panel). β-actin and GAPDH mRNA were used as internal controls for standardization. Data are mean ± SE. *n* = 4. *P < 0.05. **C.** MDA-MB-231 and MCF7 cells were lentivirally transduced with empty vector or vector encoding HOXC8, and cell lysates were subjected to immunoblotting to detect embigin (EMB), HOXC8, and β-actin. **D.** MDA-MB-231 and MCF7 cells were lentivirally transduced with vector encoding scramble sequence, or HOXC8 shRNAs, and cell lysates were subjected to immunoblotting to detect embigin (EMB), HOXC8, and β-actin.

Previously we reported that HOXC8 plays an important role in breast tumorigenesis, and acts as a transcription factor in cadherin-11 transcription in breast cancer cells [10, 19]. As a member of homeobox (HOX) protein family, it has been reported that HOXC8 regulated a number of genes involved in cell proliferation, apoptosis, migration and differentiation, etc [7, 15, 19, 20]. Among those genes, we are interested in examining whether HOXC8 is involved in regulating embigin expression in breast cancer cells. To determine the roles of HOXC8, we ectopically expressed HOXC8, or depleted HOXC8 expression by shRNA knockdown in breast cancer MDA-MB-231 and MCF7 cell lines. qRT-PCR experiments showed that HOXC8 ecto-expression decreased embigin mRNA levels, while HOXC8 knockdown increased embigin mRNA levels in both cell lines (Figure 1A, 1B). Western blotting further showed that the levels of embigin protein were decreased by HOXC8 ecto-expression, and elevated by HOXC8 shRNA knockdown (Figure 1C, 1D). We further examined the regulation of embigin expression in other breast cancer cell lines, and observed the same results: in breast cancer cell lines Hs578T and T47D, HOXC8 ecto-expression decreased both embigin mRNA and protein levels, and HOXC8 knockdown increased both embigin mRNA and protein levels (Figure S1). Taken together, these data show that HOXC8 is involved in regulation of embigin expression in breast cancer cells.

**Figure 2:**
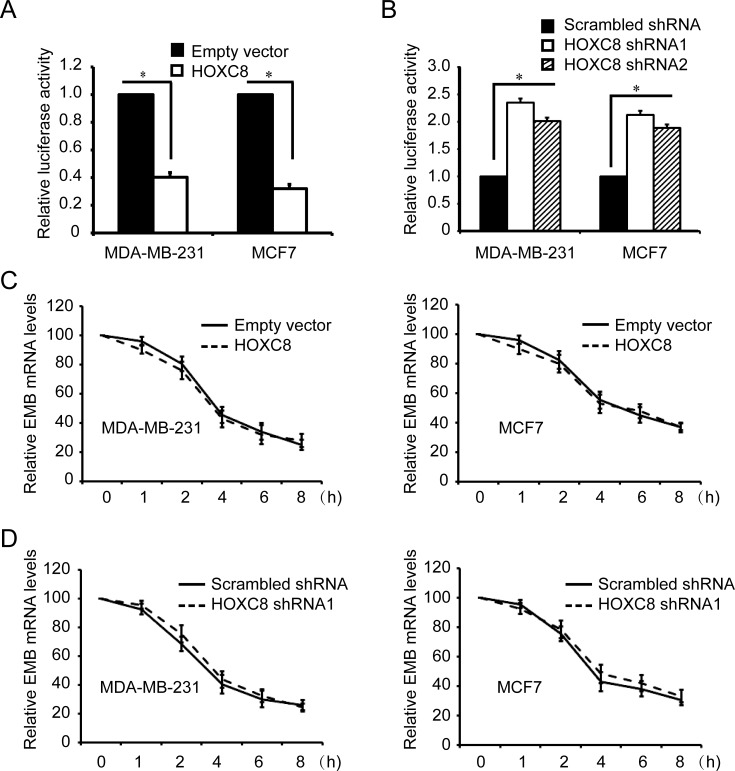
HOXC8 is involved in embigin transcription in MDA-MB-231 and MCF7 cells **A.** MDA-MB-231 and MCF7 cells were lentivirally transduced with empty vector or vector encoding HOXC8, and then transfected with EMB promoter luciferase reporter vectors that were generated using PCR amplification. Luciferase activity was measured 24h posttransfection and normalized using Renilla activities. Columns, mean; bars, SEM; *, *P* < 0.05. **B.** MDA-MB-231 and MCF7 cells were lentivirally transduced with scrambled shRNA or HOXC8 shRNA, and then transfected with EMB promoter luciferase reporter vectors along with Renilla for normalization. Luciferase activity was measured 24h posttransfection. Columns, mean; bars, SEM; *, *P* < 0.05. **C.** MDA-MB-231 or MCF7 cells were lentivirally transduced with empty vector or HOXC8 expression vector, and then were treated with 2μg/ml actinomycin for different time. Total RNA was isolated and subjected to qRT-PCR to measure the level of EMB mRNA. β-actin and GAPDH mRNA were used as internal controls. The level of EMB mRNA without actinomycin treatment was considered as 100%. Values are means ± SEM; *n* = 3. **D.** MDA-MB-231 or MCF7 cells transfected with scrambled or HOXC8 shRNA were treated 2μg/ml actinomycin. Total RNA was isolated at varying times and then subjected to qRT-PCR to measure the level of EMB mRNA. GAPDH and β-actin mRNA were used as internal controls. The level of EMB mRNA without actinomycin treatment was considered as 100%. Values are means ± SEM; *n* = 3.

Since all HOX proteins function as transcription factors, we hypothesize that embigin is probably one of target genes of HOXC8. To test this hypothesis, we generated embigin promoter reporter plasmid by subcloning embigin promoter region into firefly luciferase reporter vector (Figure S2) [21]. Analyzing with this plasmid, we found that HOXC8 expression significantly inhibited the luciferase activities of embigin promoter, while HOXC8 knockdown by shRNA transduction increased its luciferase activities in both MDA-MB-231 and MCF7 cells (Figure 2A, 2B; Figure S3). To further determine whether HOXC8 is involved in embigin transcription, we performed actinomycin-chasing experiments and found that HOXC8 ecto-expression or shRNA knockdown did not affect embigin mRNA stability (Figure 2C, 2D). These data indicate that HOXC8 regulates embigin transcription in breast cancer cells.

### HOXC8 binds to embigin promoter

**Figure 3:**
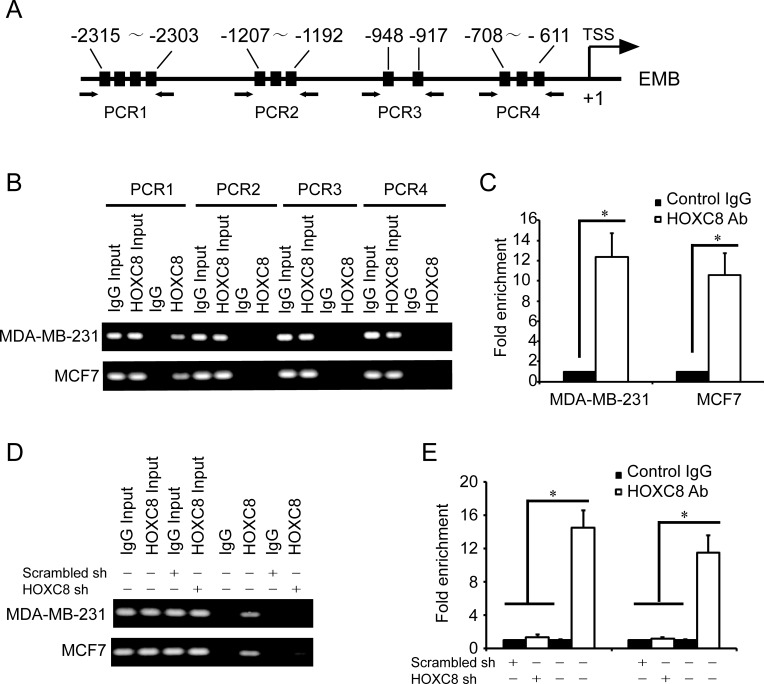
HOXC8 binds to the promoter of EMB **A.** A schematic diagram for the positions of putative HOX protein binding sites in the promoter of EMB. Arrows show the regions for PCR primers amplification. **B.** ChIP was performed on MDA-MB-231 or MCF7 cells using HOXC8 antibody or mouse IgG and the immunoprecipitated chromatin DNA was subjected to PCR using primers to amplify the region of EMB promoter. **C.** ChIP was performed on MDA-MB-231 or MCF7 cells using HOXC8 antibody or IgG as control, and precipitated chromatin DNA were subjected to qPCR using primers to amplify the regions of EMB promoter. **D.** MDA-MB-231 and MCF7 cells were lentivirally transduced with HOXC8 shRNA vectors or scrambled shRNA vectors, and HOXC8 ChIP assays were performed with IgG as negative control. **E.** MDA-MB-231 and MCF7 cells were lentivirally transduced with HOXC8 shRNA vectors or scrambled shRNA vectors, and HOXC8 ChIP assays were performed with IgG as negative control. Precipitated chromatin DNA were subjected to qPCR using primers to amplify the regions of EMB promoter.

To confirm the roles of HOXC8 in embigin transcription, we examined whether HOXC8 binds to embigin promoter by ChIP assay. Based on the known HOX protein-binding sequences [22], there are several HOX potential binding sites that locate in four regions within embigin promoter, so we designed several sets of primers that specifically amplify these regions (Figure 3A). HOXC8 ChIP assay showed that HOXC8 binds to the region of embigin promoter from nucleotides −2303 to −2315 (Figure 3B), but not to the other three regions in EMB promoter. Quantitative PCR (qPCR) further demonstrated that the region of −2303 to −2315 was enriched 10-12 fold in the HOXC8 chromatin immunoprecipitates in both MDA-MB-231 and MCF7 cells compared to IgG control (Figure 3C). The above results were further confirmed by HOXC8 ChIP assay performed in HOXC8 knockdown cells or control cells, in which HOXC8 knockdown abolished the recruitment of HOXC8 to EMB promoter (Figure 3D, 3E). These data suggest that HOXC8 regulates embigin expression by binding to embigin promoter at the region from nucleotide −2303 to −2315 in MDA-MB-231 and MCF7 cells.

### HOXC8-embigin pathway is involved in proliferation, anchorage-independent cell growth and migration of breast cancer cells

**Figure 4:**
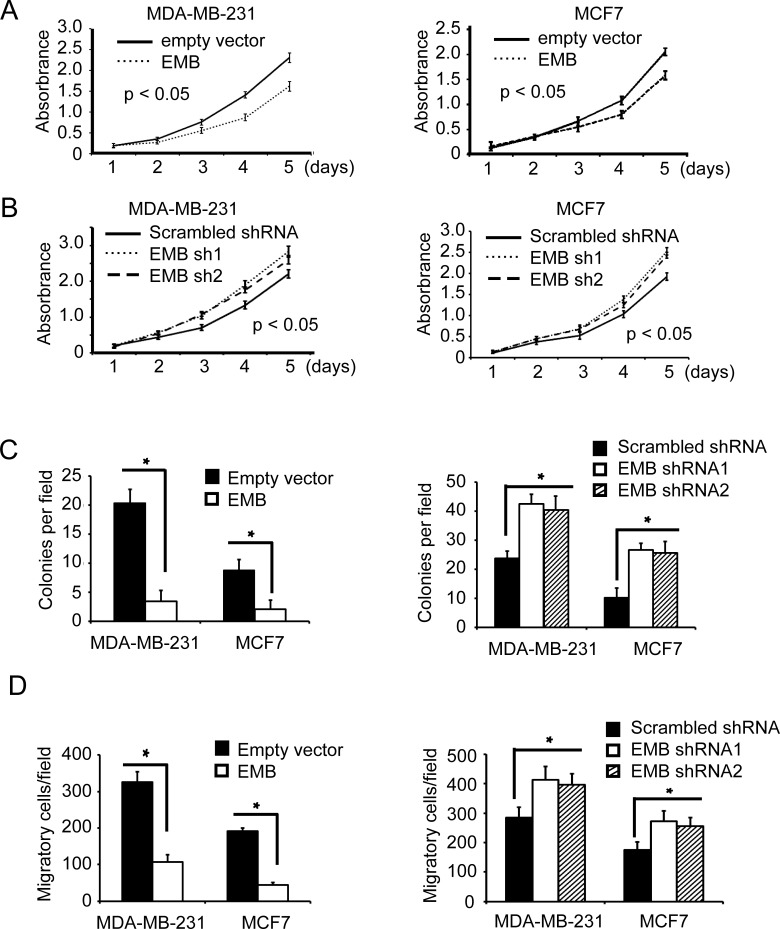
Embigin suppresses proliferation, anchorage-independent cell growth and migration of breast cancer cells **A.** MTT assay to analyze cell proliferation of MDA-MB-231 and MCF7 cells that were lentivirally transduced with empty vectors or EMB expression vectors. Data are the mean ±SE. *n* = 3. **B.** MTT assay to analyze cell proliferation of MDA-MB-231 and MCF7 cells that were lentivirally transduced with scrambled shRNA or EMB shRNA vectors. Data are the mean ±SE. *n* = 3. **C.** Soft agar assay to analyze cell anchorage-independent cell growth of MDA-MB-231 and MCF7 that were transduced with EMB expression vectors (left panel) or EMB shRNA vectors (right panel). Data are the mean ±SE. *n* = 3. *, *P* < 0.05. **D.** Transwell assay to analyze cell migration of MDA-MB-231 and MCF7 that were transduced with EMB expression vectors (left panel) or EMB shRNA vectors (right panel). Data are the mean ±SE. *n* = 3, *, *P* < 0.05.

Embigin is expressed in regressing prostate and mammary gland and also detected in prostate and mammary cancer cell lines [4], but very little is known about its function in breast cancer cells. To determine the function of embigin in breast cancer cells, we analyzed the effect of embigin on cell proliferation, anchorage-independent cell growth and cell migration in MDA-MB-231 and MCF7 cells. In both cell lines, MTT assay showed that embigin knockdown significantly enhanced cell proliferation, and embigin ecto-expression decreased cell proliferation compared to control cells (Figure 4A, 4B; Figure S4, Figure S5). In soft-agar colony formation assay, we found that ecto-expression of embigin significantly suppressed anchorage-independent cell growth of both MDA-MB-231 and MCF7 cells, while embigin knockdown significantly enhanced anchorage-independent cell growth (Figure 4C, Figure S6). Furthermore, transwell assay showed that embigin overexpression inhibited cell migration, and embigin knockdown enhanced the migration of breast cancer cells (Figure 4D, Figure S7). These data indicate that embigin plays a suppressive role in breast tumor proliferation, anchorage-independent growth and migration.

**Figure 5:**
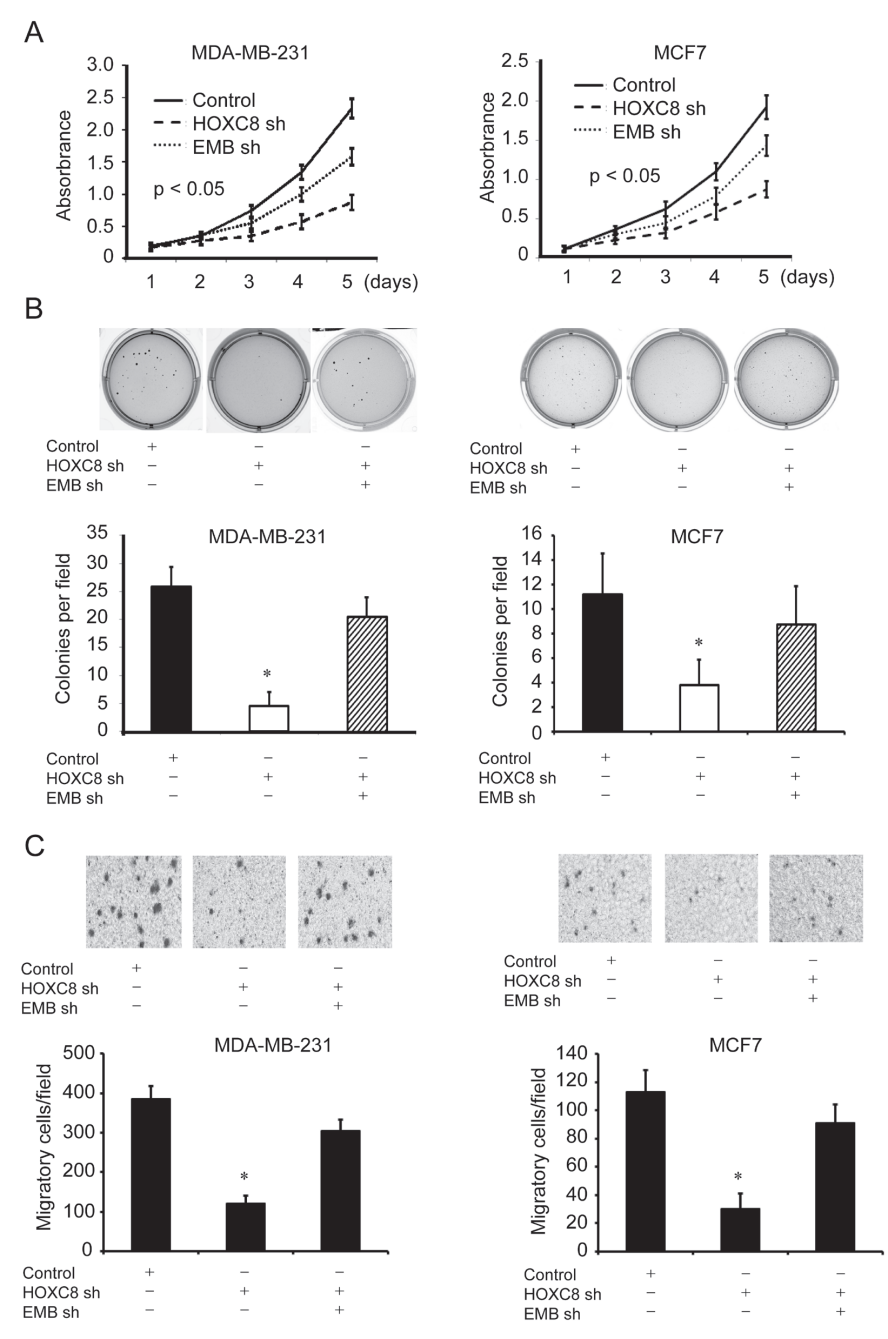
Embigin depletion reverses the inhibitory effects of HOXC8 knockdown on proliferation, anchorage-independent cell growth, cell migration of breast cancer cells **A.** HOXC8 knockdown MDA-MB-231 or MCF7 cells were transduced with scrambled or embigin shRNA vectors, and MTT assays were performed to analyze cell proliferation. Data are the mean ±SE. *n* = 3. **B.** HOXC8 knockdown MDA-MB-231 or MCF7 cells were transduced with scrambled or embigin shRNA vectors, and soft agar assays were performed to analyze cell anchorage-independent growth. Data are the mean ±SE. *n* = 3. *, *P* < 0.05. **C.** HOXC8 knockdown MDA-MB-231 or MCF7 cells were transduced with scrambled or embigin shRNA vectors, and transwell assays were performed to analyze cell migration. Data are the mean ±SE. *n* = 3. *, *P* < 0.05.

Previously we reported that HOXC8 mediates proliferation, migration and metastasis of breast cancer cells, it is interesting to explore whether HOXC8 affects these behaviors by regulating embigin transcription. To investigate the functional link between HOXC8 and embigin expression, MDA-MB-231 or MCF7 of HOXC8 knockdown cells were transduced with embigin shRNA lentiviral vectors, and then examined with MTT, soft-agar or migration assays. As shown in Figure 5. HOXC8-knockdown cells displayed impaired capability in cell proliferation, anchorage-independent cell growth and cell migration, which can be largely recovered by embigin knockdown in both MDA-MB-231 and MCF7 cells (Figure 5A, 5B & 5C). Taken together, these results show that inhibitory effects of embigin on cell proliferation, anchorage-independent growth and cell migration are functionally regulated, at least in part, by HOXC8 protein in breast cancer cells.

### EMB knockdown enhances tumorigenesis capability of normal breast cell line MCF10A

**Figure 6:**
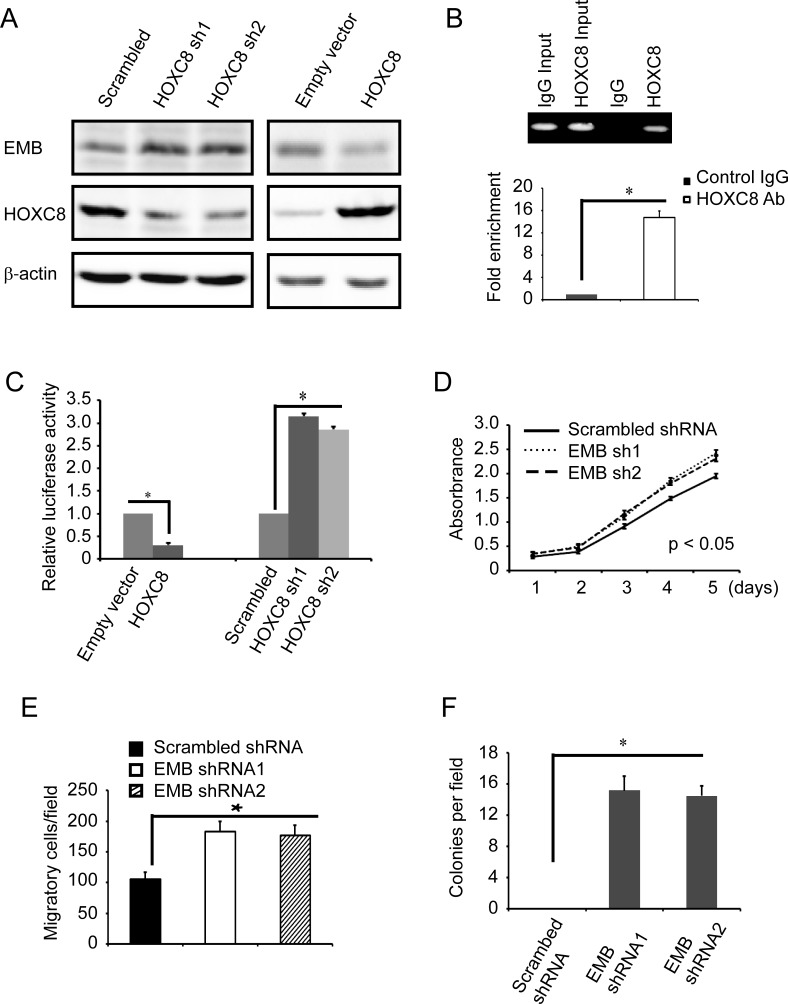
Embigin knockdown stimulates proliferation, anchorage-independent growth and migration of MCF10A cells **A.** MCF10A cells were lentivirally transduced with HOXC8 expression vectors or HOXC8 shRNA vectors, and cell lysates were subjected to immunoblotting to detect embigin (EMB), HOXC8, and β-actin. **B.** ChIP was performed on MCF10A cells using HOXC8 antibody or mouse IgG and the immunoprecipitated chromatin DNA was subjected to PCR (upper panel) or real-time PCR (lower panel). **C.** MCF10A cells were lentivirally transduced with HOXC8 expression vectors or HOXC8 shRNA vectors, and then transfected with EMB promoter luciferase reporter vectors. Luciferase activity was measured 24h posttransfection and normalized using Renilla activities. Columns, mean; bars, SEM; *, *P* < 0.05. **D.** MTT assay to analyze proliferation of MCF10A cells that were lentivirally transduced with scrambled shRNA or EMB shRNA vectors. Data are the mean ±SE. *n* = 3. **E.** Transwell assay to analyze migration of MCF10A cells that were lentivirally transduced with scrambled shRNA or EMB shRNA vectors. Data are the mean ±SE. *n* = 3. *, *P* < 0.05. **F.** Soft agar assay to analyze colony formation of MCF10A cells that were lentivirally transduced with scrambled shRNA or EMB shRNA vectors. Data are the mean ±SE. *n* = 3. *, *P* < 0.05.

To further explore the roles of embigin in breast tumorigenesis, we investigated the effects of embigin silencing on the proliferation, anchorage-independent growth and migration capability in normal epithelial breast cell line MCF10A. We first examined whether HOXC8 is involved in EMB transcription regulation in MCF10A cells by HOXC8 ecto-expression or HOXC8 shRNA knockdown. We observed that HOXC8 expression significantly decreased both embigin protein and mRNA levels, while HOXC8 knockdown increased both embigin protein and mRNA levels in MCF10A cells (Figure 6A, Figure S8). ChIP assay in MCF10A cells showed that HOXC8 binds to the region of embigin promoter from nucleotides −2303 to −2315, and the region of embigin promoter is enriched more than 12 folds in HOXC8 immunoprecipitates compared to IgG control (Figure 6B). Luciferase assay further demonstrated that HOXC8 expression decreases embigin promoter activities, and HOXC8 knockdown increases embigin promoter activities, indicating that HOXC8 inhibits embigin transcription in MCF10A cells (Figure 6C). Taken together, these data indicated that HOXC8 also functions as a transcription inhibitor for embigin gene in MCF10A cells.

We subsequently investigated the roles of embigin by examining the proliferation, anchorage-independent growth and migration in embigin knockdown MCF10A cells. As shown in Figure 6, we observed that depletion of embigin results in increased proliferation, colony formation and migration in MCF10A cells transduced with embigin shRNA knockdown vectors, compared to MCF10A cells transduced with scrambled shRNA vectors (Figure 6D, 6E & 6F; Figure S9). These data indicated that loss of embigin enhances the tumorigenesis capability of normal epithelia breast cancer cell MCF10A.

### Loss of embigin correlates with poor survival of breast cancer patients

**Figure 7:**
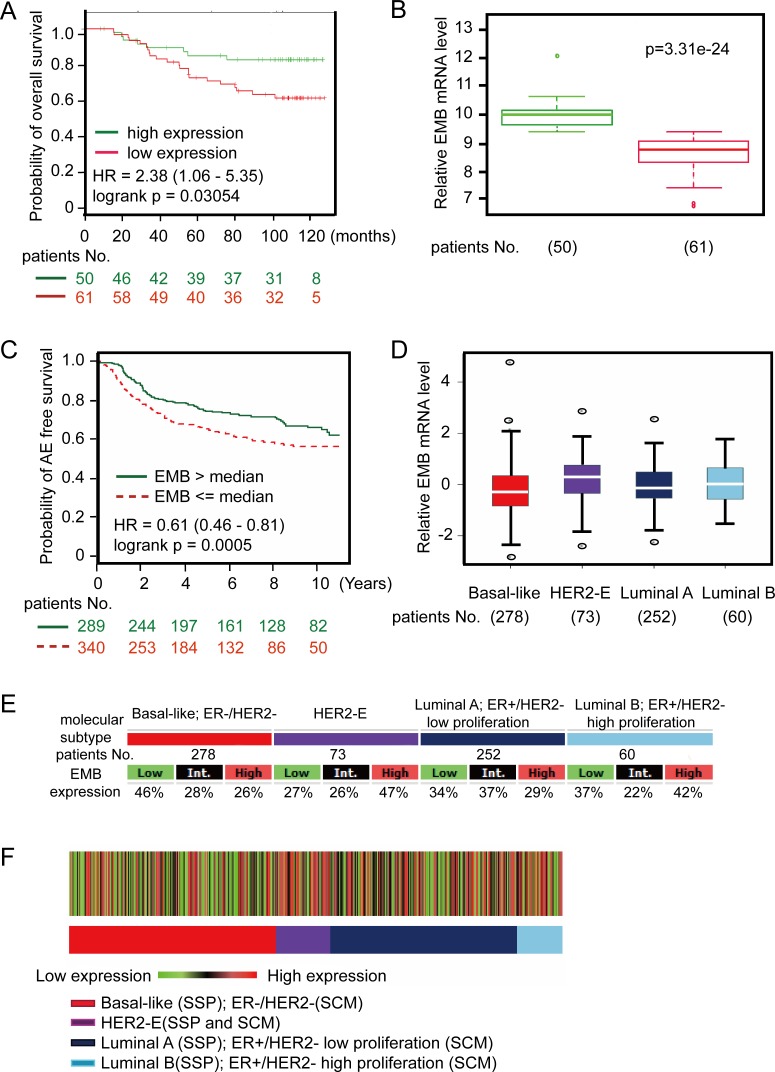
Embigin expression is low in basal-like breast cancer clinical samples and are negatively associated with survival of patients with basal-like cancers **A.** Kaplan-Meier curve showing overall survival of breast cancer patients with high or low embigin expression (logrank *p* = 0.003054). Data were analyzed using SurvExpress web program with GSE19536, and the patient samples were split into two groups according prognostic index (PI). **B.** Box plot shows embigin expression between high risk group (red, 61 patients) and low risk group (green, 50 patients). Data were analyzed using SurvExpress web program with GSE19536. **C.** Kaplan-Meier curve showing any-event free survival of basal-like tumors with high or low embigin expression (logrank *p* = 0.0005). Data were analyzed using bc-GenExMiner web application, and the patient samples (629 samples) were split into two groups according to median expression of embigin (high expression group: 289 samples; low expression group: 340 samples). **D.** The graph shows embigin expression levels on breast cancer subtype with patients No. indicated. **E.** Embigin expression table. For each breast cancer subtype, the number of samples and the percentages of tumors with low, intermediate, or high embigin mRNA expression are indicated. **F.** Heatmap of embigin gene expression on patients based on robust single sample predictor (SSP) classification with the three SSPs. Percentage of tumors with high, intermediate, and low embigin expression per molecular subtype are given in the gene expression table.

The results described above pointed out that HOXC8 regulates embigin transcription to mediate breast tumor progression. Previously we demonstrated that high expression of HOXC8 was significantly associated with low recurrence-free survival rate of breast cancer patients, so we evaluated the correlation between embigin expression and breast tumor recurrence. Using web-based survival analysis applications [16–18], we examined the association between embigin expression and patients survival probability, which showed that the patients with low embigin expression have decreased survival probability compared to patients with high embigin expression (Figure 7A, 7B, log rank *p* = 0.003045; Figure S10, log rank *p* = 0.001). To validate the roles of embigin in breast cancer progression, we further analyzed embigin expression in different breast cancer subtypes [17]. As shown in Figure 7C, low embigin expression was significantly associated with decreased survival rate in patients with basal-like cancers (logrank *p* = 0.0005). Notably, molecular subtype association analysis of transcriptional profiles from clinical breast cancer samples revealed that embigin expression was low specifically in basal-like, ER-/HER2- patients, in which 46% basal-like tumors expressed the lowest levels of embigin consistent with a potential role for embigin in this subtype (Figure 7D, 7E & 7F). Taken together, these data suggest that embigin loss promotes breast cancer development, and may play a particularly important role in the progression of basal-like tumors.

## DISCUSSION

The present study shows, for the first time, that embigin plays a suppressive role in breast cancer progression, particularly in basal-like tumors. We initially demonstrated that HOXC8 functions as a transcription factor to inhibit embigin transcription, and embigin knockdown enhances the proliferation, anchorage-independent growth and migration of breast cancer cells and normal breast cells. Moreover, we found that HOXC8 mediates breast tumorigenesis by, at least partially, regulating embigin expression in breast cancer cells. Finally, online data analysis showed that embigin expression is low in basal-like tumors and loss/low of embigin significantly correlates with increased tumor recurrence in patients, particularly those with basal-like cancers.

HOXC8 protein expression was found to be deregulated in various cancers [11–14, 23]. Although it remains unclear how HOXC8 takes part in cancer process, HOXC8 must act as a transcription factor to regulate its target genes that participate in biological processes. The HOX proteins all contain a highly conserved 60-amino-acid motif, the homeodomain, which is responsible for binding to DNA of their target genes. Recent studies revealed a core DNA-binding sequence (TAATNN) for HOX proteins binding [22, 24]. It has been reported that HOXC8 is involved in regulation of a number of genes expression, including embigin gene [7, 15]. Therefore, we analyzed the promoter of embigin for potential HOX protein binding sites, and performed luciferase assay and ChIP assay to determine HOXC8 binding sites on embigin promoter. We demonstrated that HOXC8 binds to embigin promoter at the region from nucleotides −2303 to −2315 and inhibits embigin transcription. More importantly, depletion of HOXC8 leads to significant reduction of proliferation, colony formation and migration of breast cancer cells, which can be largely recovered by embigin knockdown, suggesting the functional linking between HOXC8 and embigin in breast cancer cells. These data indicated that HOXC8 functions as a transcription factor to inhibit embigin expression in breast cancer cells. It has been reported that HOXC8 takes part in a number of genes regulation in mouse fibroblasts[7, 15], and our previous study also showed that HOXC8 promotes breast cancer cell migration and metastasis through activating cadherin-11 transcription[11, 19]. Based on these findings, we hypothesize that HOXC8 regulates various genes transcription in breast cancer development, which includes activating cadherin-11 and inhibiting embigin transcription in breast cancer cells.

ChIP assay shows that HOXC8 binds to EMB promoter at the region from nucleotides −2303 to −2315, however, there are several putative HOX binding sites in this region, and there are other three regions of embigin promoter containing HOX binding sites, too (Figure 3A), suggesting that other HOX proteins may also take part in the regulation of embigin transcription in breast cancer cells. For instance, one well-identified HOXC8 target gene is osteopontin (OPN), which is found to be regulated by both HOXC8 and HOXA9. Studies showed that both HOXC8 and HOXA9 act as transcription factor to bind to the promoter of osteopontin, where HOXC8 formed heterodimers with Smad1 and HOXA9 formed heterodimers with Smad4 [25]. In our previous study, we showed evidences that HOXC8 functions as transcription activator to induce cadherin-11 expression in breast cancer cells [11, 19], which suggested that HOXC8 may associate with different co-factors to activate or inhibit its target genes transcription in breast cancer cells. Therefore, further study is awaited to determine whether HOXC8 co-operates with other co-factors to regulate embigin transcription.

Embigin is a glycoprotein belonging to the immunoglobulin superfamily, and is strongly expressed in the endoderm and then undetectable after 10 days of gestation during embryogenesis. Embigin is expressed in various tissues, and probably takes part in tissue reorganization. Embigin is also detected in cancer cell lines, such as breast cancer cells MDA-MB-231 and MCF7, and its expression is decreased upon culture in Matrigel [4], indicating embigin may play a role in breast tumorigenesis. Consistent with these data, we found that depletion of embigin expression resulted in increased proliferation, anchorage-independent growth and migration of MDA-MB-231 and MCF7 cells. In normal breast epithelial cell MCF10A, embigin knockdown also leads to increase cell proliferation, anchorage-independent growth and migration. Moreover, publically available microarray data shows that embigin expression is low in breast basal-like tumors, and low embigin expression is significantly associated with decreased survival rate in patients with basal-like, the most aggressive subtype of human breast cancer, underscoring the connection between embigin loss and breast cancer progression. Our data indicate that embigin could be useful as a prognostic biomarker in breast cancers, at least the subset of breast basal-like cancers, although our observations do not preclude its potential involvement in other subtypes. In conclusion, this study demonstrates that embigin is transcriptionally regulated by HOXC8 protein and its low/loss expression may play an important role in the progression of breast cancers.

## MATERIALS AND METHODS

### Cells and materials

The human breast tumor cell lines were obtained from American Type Culture Collection (ATCC) and cultured in the medium according to ATCC protocol. Anti-HOXC8 rabbit polyclonal antibody (titer, 1:1000) was from Sigma. Anti-embigin pAB(titer, 1:1,000) and anti-β-actin mAb (titer, 1:1,000) were obtained from Santa Cruz Biotechnology. TRIzol RNA extraction reagent, Lipofectamine 2000 & LTX were purchased from Life Technologies. ECL SuperSignal West Femto Maximum Sensitivity Substrate was purchased from Thermo Scientific. Chemicals and other materials were purchased from Sigma or Life Technologies.

### Construction of shRNA and gene expression lentiviral vectors

Embigin or HOXC8 shRNA sequences were generated with the aid of web-based Invitrogen Block-It program and expressed from pLV-shRNA vector (BioSettia, San Diego, CA). Embigin lentiviral expression vector was developed by subcloning human embigin cDNA into pCDH-CMV-MCS-EF1-Puro (System Biosciences, Mountain View, CA). Lentiviruses were prepared as previously described [10]. All related sequences are included in Supplemental Table S1.

### Promoter reporters and luciferase assay

The full length Embigin promoter [21] was amplified from genomic DNA of MDA-MB-231 cells(Figure S1) and the fragment was subcloned into the luciferase reporter plasmids pGL4.23 vector (Promega). PCR products were verified by DNA sequencing. For luciferase assay, cells were cultured without antibiotics overnight in 24-well plates and then transfected with embigin promoter reporter plasmids using lipofectamin LTX (Invitrogen). After 24 hours, cells were washed with phosphate-buffered saline (PBS), subjected to lysis, and luciferase activities were measured using the dual luciferase assay kit (Promega). Three independent experiments were performed in triplicate.

### Chromatin Immunoprecipitation (ChIP) assay

ChIP assays were carried out as described previously [18]. Briefly, MDA-MB-231, MCF7 or MCF10A cells were grown to near confluency in 15cm dishes. Cells were fixed in 1% formaldehyde; sheared chromatin was prepared, precleared with protein G-agarose, and immunoprecipitated with anti-HOXC8 antibody overnight at 4°C. Immune complexes were captured using protein G-agarose, and the formaldehyde cross-links in the eluted complexes were reversed. The DNA was analyzed by PCR or real-time PCR. All related sequences are included in Supplemental Table S1.

### Western blotting

Cell lysates were subjected to SDS-PAGE and electroblotted onto PVDF membranes. Immunoblottings were performed as previously described [10].

### Quantitative reverse transcriptase-PCR (qRT-PCR)

Total RNA was extracted from cells using Trizol, treated by DNase I and reverse transcribed with SuperScriptase (Life Technologies). Generated products were applied to ABI 7900 real-time PCR system with specific primers. Level of β-actin and GAPDH mRNA were measured for standardization.

### MTT cell proliferation assay

Cell proliferation was evaluated by MTT assay. Briefly, 5 × 10^4^ cells were seeded into 24-well culture plates and allowed to grow for different days before the addition of MTT. The cell growth rate was determined by reading plates at 560 nm.

### Soft agar colony formation assay

MDA-MB-231, MCF7 or MCF10A cells were seeded for colony formation in 6-well plates at 2 × 10^4^ cells per well. Each well consisted of a bottom base layer (0.6% agarose diluted in DMEM) and top layer (0.3% agarose diluted in DMEM). We added a few drops of DMEM to the solidified top layer. The top layer was replenished on a weekly basis. After 3-4 weeks, colonies were stained with iodonitrotetrazolium chloride (INT) and counted under a phase-contrast microscope. Each assay was performed in triplicate.

### Transwell migration assay

Cell migration was performed as previously described [10]. Briefly, the undersurface of transwells (8 μm pore size; Costar) was coated with 10 μg/mL of collagen I overnight at 4°C. Cells were detached with 0.05% Trypsin, and suspended in serum-free medium at a density of 5 × 10^6^ cells/mL. 100 μL of the cell suspension was added to the upper chamber of the transwells, and DMEM medium with 10%FBS was added into lower chamber as attractants. After a 4-hour migration period, the remaining cells in the upper chamber were removed with cotton swabs and the cells on the undersurface of transwells were fixed with 5% glutaraldehyde solution. Cells were stained with crystal violet solution and the number of migratory cells was calculated by counting three different fields under a phase-contrast microscope.

### Bioinformatics analysis

Gene expression data and survival analysis were performed with publically available data using bc-GenExMiner, SurvExpress and PROGgene web-based tools as described [16–18]. To analyze the prognostic value of embigin, Kaplan-Meier method was used to estimate survival curves and the log-rank test was used to compare survival curves of high and low embigin expression groups. All analyses were performed following instructions of web applications. Survival analysis with SurvExpress was performed using GSE19536 dataset (111 patient samples), and the patient samples were split into two groups according prognostic index (PI). Survival analysis with PROGgene was performed using GSE19615 dataset (114 patient samples), and the patient samples were split into two group according to median expression of embigin. The survival analysis with bc-GenExMiner was performed using the pooled microarray dataset (629 patient samples), and the patient samples were split into two group according to median expression of embigin. Survival information and embigin gene expression was further analyzed based on molecular subtype predictors (single sample predictors, SSPs) using bc-GenExMiner. For all statistical analyses, *p* < 0.05 were considered as significant.

### Statistical analysis

All data are presented as means and s.e.m. statistical analyses were performed on data collected from at least three independent experiments. Student's t-test (two-tailed) was used to compare two groups, and differences were considered statistically significant when *P* < 0.05.

## SUPPLEMENTARY MATERIAL FIGURES AND TABLE


