# Supplementary material for: Embigin, regulated by HOXC8, plays a suppressive role in breast tumorigenesis
Source: Oncotarget. 2015 Jun 8;6(27):23496–509. doi: 10.18632/oncotarget.4360 (PMC4695132; doi:10.18632/oncotarget.4360)
Supplement: Supplementary file 1 [file oncotarget-06-23496-s001.pdf]

Embigin, regulated by HOXC8, plays a suppressive role in breast tumorigenesis

Supplementary Material

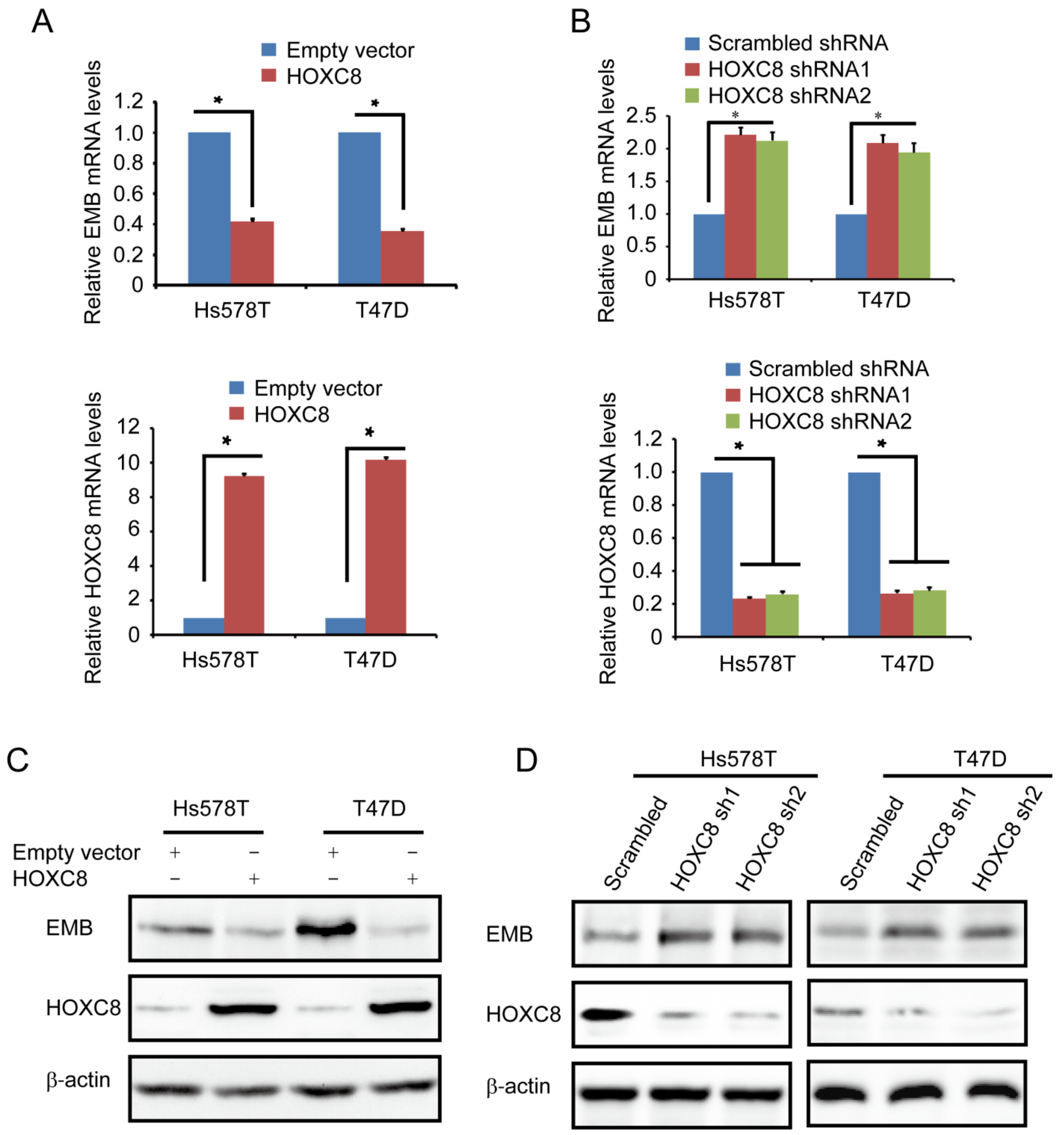

Figure S1: **HOXC8 regulates embigin expression in Hs578T and T47D cell lines.** (A) Hs578T and T47D cells were lentivirally transduced with empty vector or vector encoding

HOXC8, and total RNA from these cells was subjected to qRT-PCR to determine the level of EMB mRNA (upper panel) and the level of HOXC8 (lower panel). β-actin and GAPDH mRNA were used as internal controls for standardization. Data are mean ± SE. n = 4. *P < 0.05. (B) Hs578T and T47D cells were lentivirally transduced with vector encoding scramble sequence, or HOXC8 shRNAs. Total RNA was isolated from these cells and subjected to qRT-PCR to determine the level of EMB mRNA (upper panel) and the level of HOXC8 (lower panel). β-actin and GAPDH mRNA were used as internal controls for standardization. Data are mean ± SE. n = 4. *P < 0.05. (C) Hs578T and T47D cells were lentivirally transduced with empty vector or vector encoding HOXC8, and cell lysates were subjected to immunoblotting to detect embigin (EMB), HOXC8, and β-actin. (D) Hs578T and T47D cells were lentivirally transduced with vector encoding scramble sequence, or HOXC8 shRNAs, and cell lysates were subjected to immunoblotting to detect embigin (EMB), HOXC8, and β-actin.

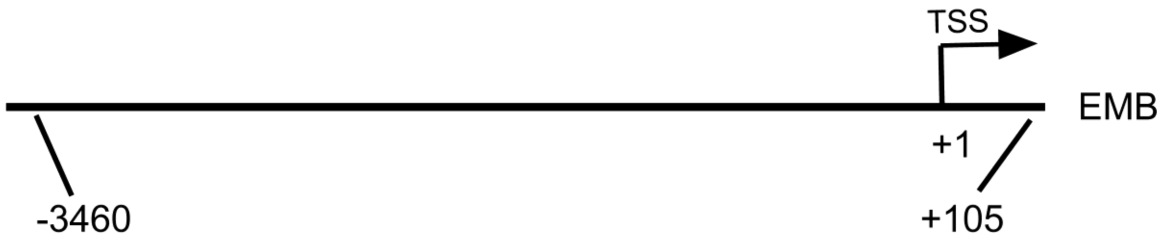

Figure S2: A schematic diagram depicts the EMB promoter for luciferase assay. Embigin promoter (nt -3460 to +105) was amplified by PCR and subcloned into luciferase reporter vector pGL4.23 (Promega).

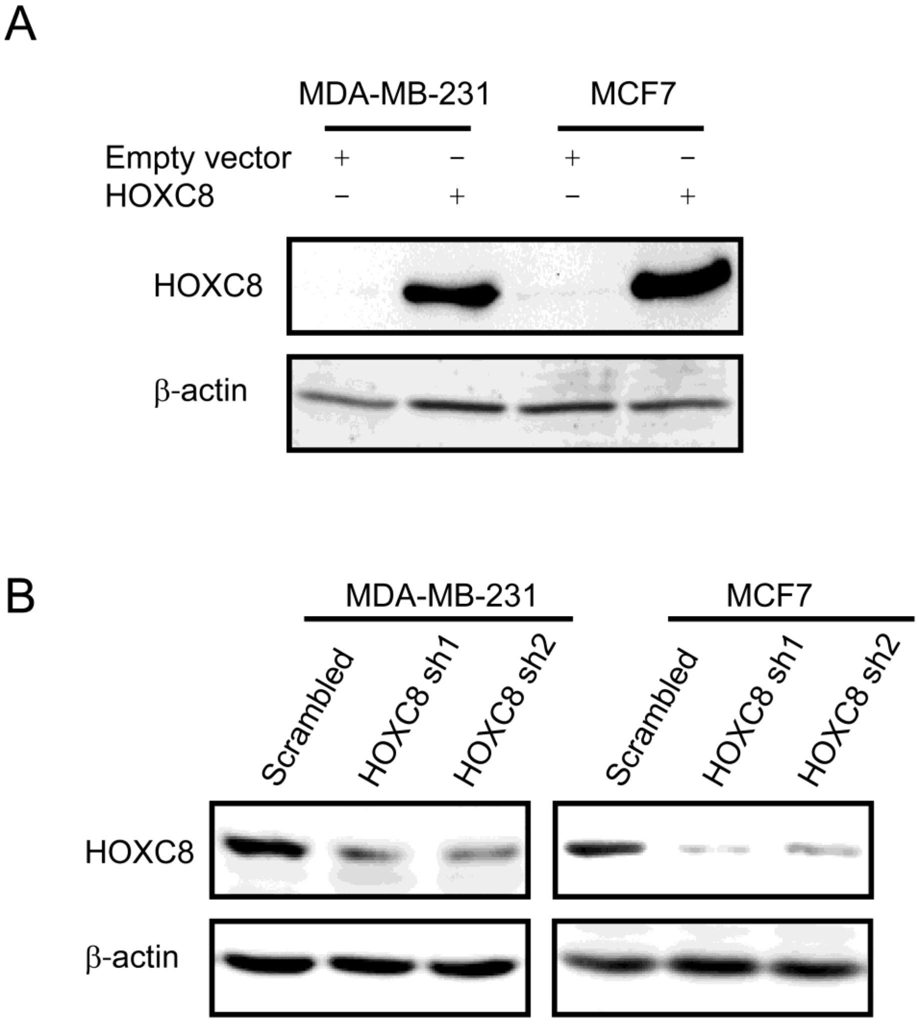

Figure S3: HOXC8 ecto-expression or shRNA knockdown in MDA-MB-231 and MCF7 cells. (A) MDA-MB-231 or MCF7 cells were lentivirally transduced with HOXC8 expression vectors or empty vectors, and cell lysates were subjected to SDS-PAGE and immunoblotted for HOXC8 and β-actin. (B) MDA-MB-231 or MCF7 cells were lentivirally transduced with scrambled shRNA or HOXC8 shRNA vectors. Cell lysates were subjected to SDS-PAGE and immunoblotted for HOXC8 and β-actin.

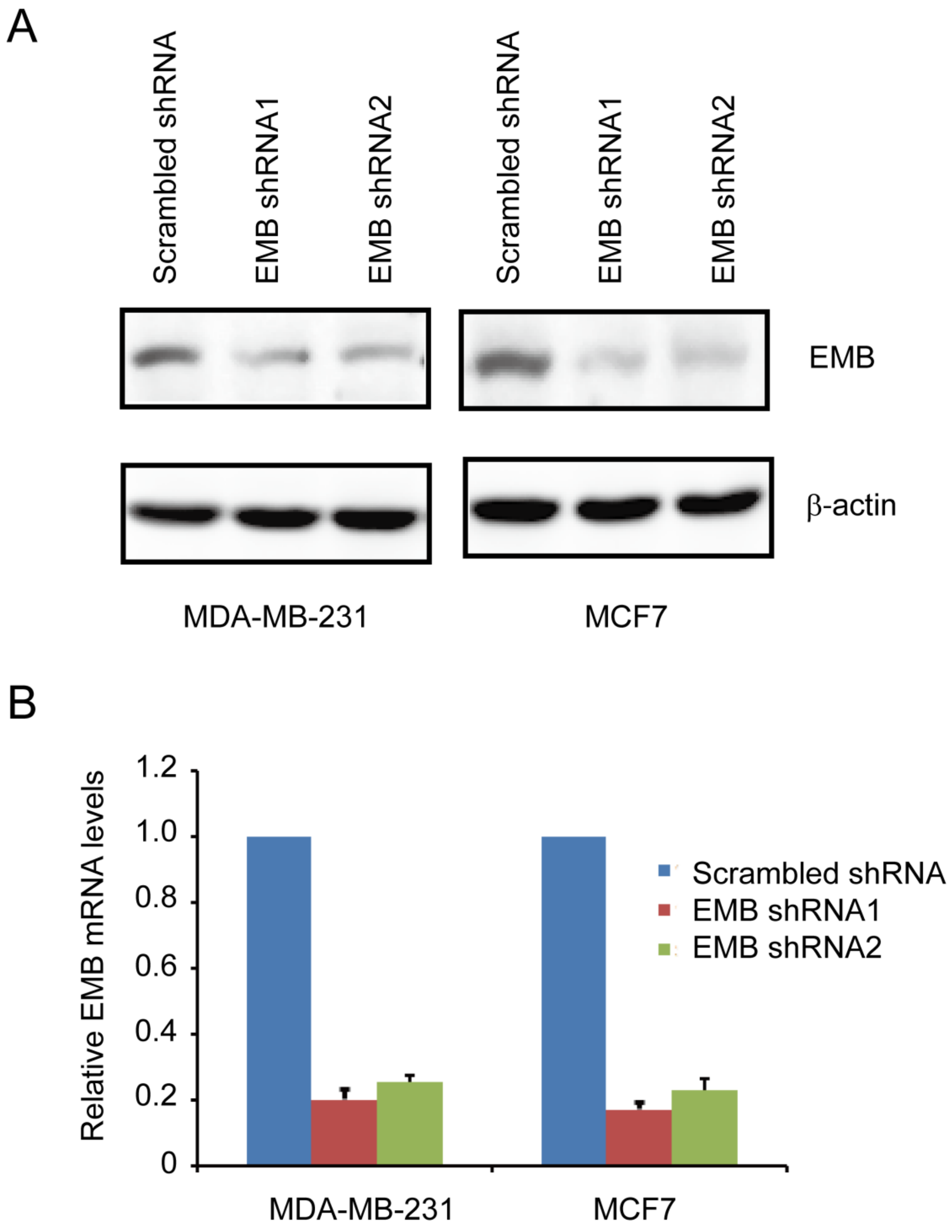

Figure S4: EMB shRNA knockdown in MDA-MB-231 and MCF7 cells. MDA-MB-231 or MCF7 cells were lentivirally transduced with scrambled shRNA or embigin shRNA vectors. (A) Cell lysates were subjected to SDS-PAGE and probed for embigin (EMB) and β-actin. (B) Total RNA isolated from EMB knockdown cells was subjected to qRT-PCR to measure the levels of EMB mRNA. Data were normalized to the levels of β-actin mRNA. Columns, mean; bars, SEM.

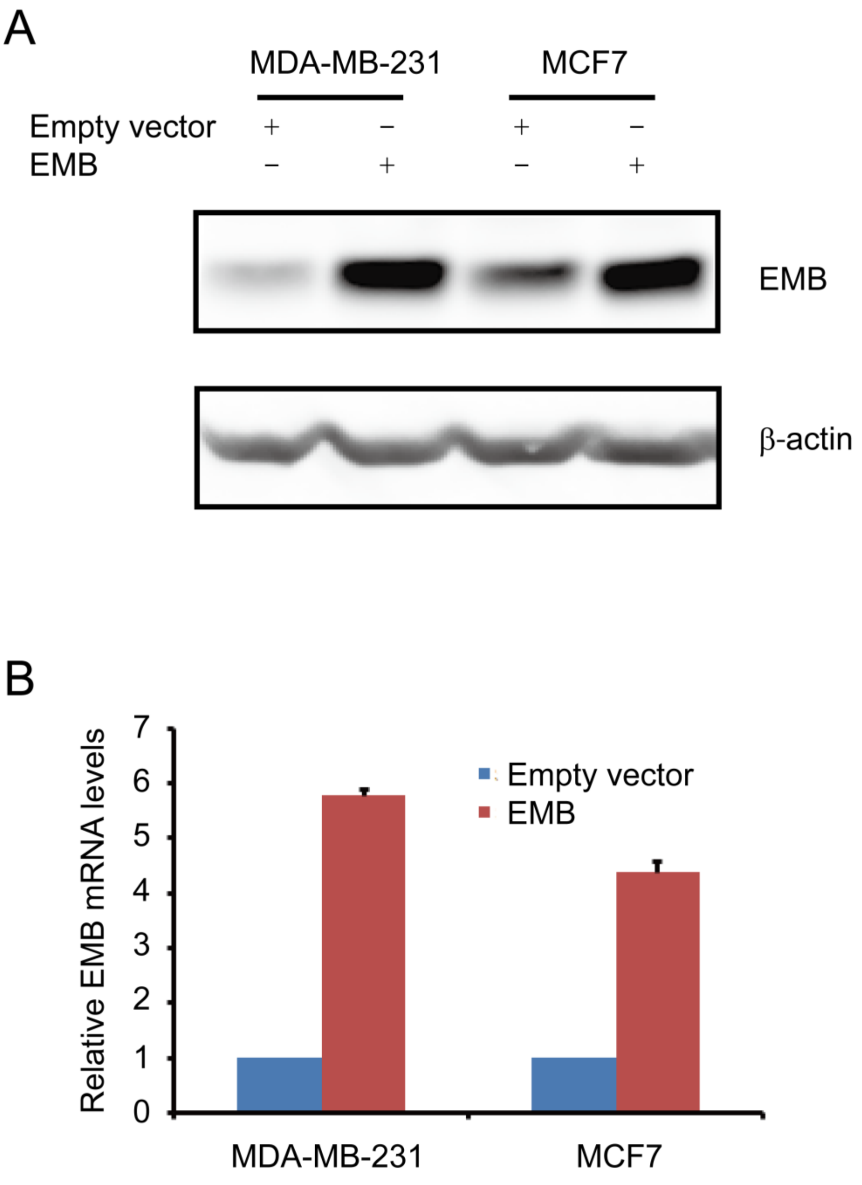

Figure S5: **EMB ecto-expression in MDA-MB-231 and MCF7 cells.** MDA-MB-231 or MCF7 cells were lentivirally transduced with empty or embigin expression vectors. (A) Cell lysates were subjected to SDS-PAGE and probed for embigin (EMB) and β-actin. (B) Total RNA was subjected to qRT-PCR to measure the levels of EMB mRNA. Data were normalized to the levels of β-actin mRNA. Columns, mean; bars, SEM.

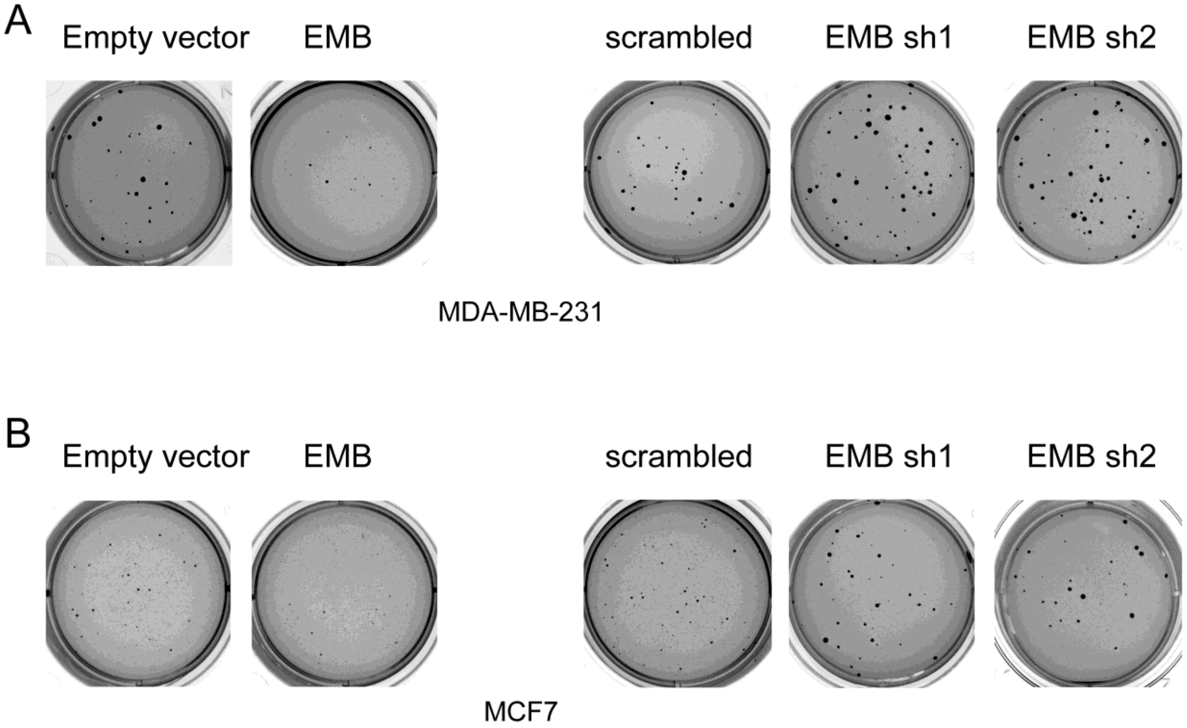

Figure S6: Soft agar assay performed in MDA-MB-231 and MCF7 cells. (A) MDA-MB-231 cells were lentivirally transduced with EMB expression vectors or shRNA vectors, and then soft agar growth was assessed and photographed. (B) MCF7 cells were lentivirally transduced with EMB expression vectors or shRNA vectors, and then soft agar growth was assessed and photographed.

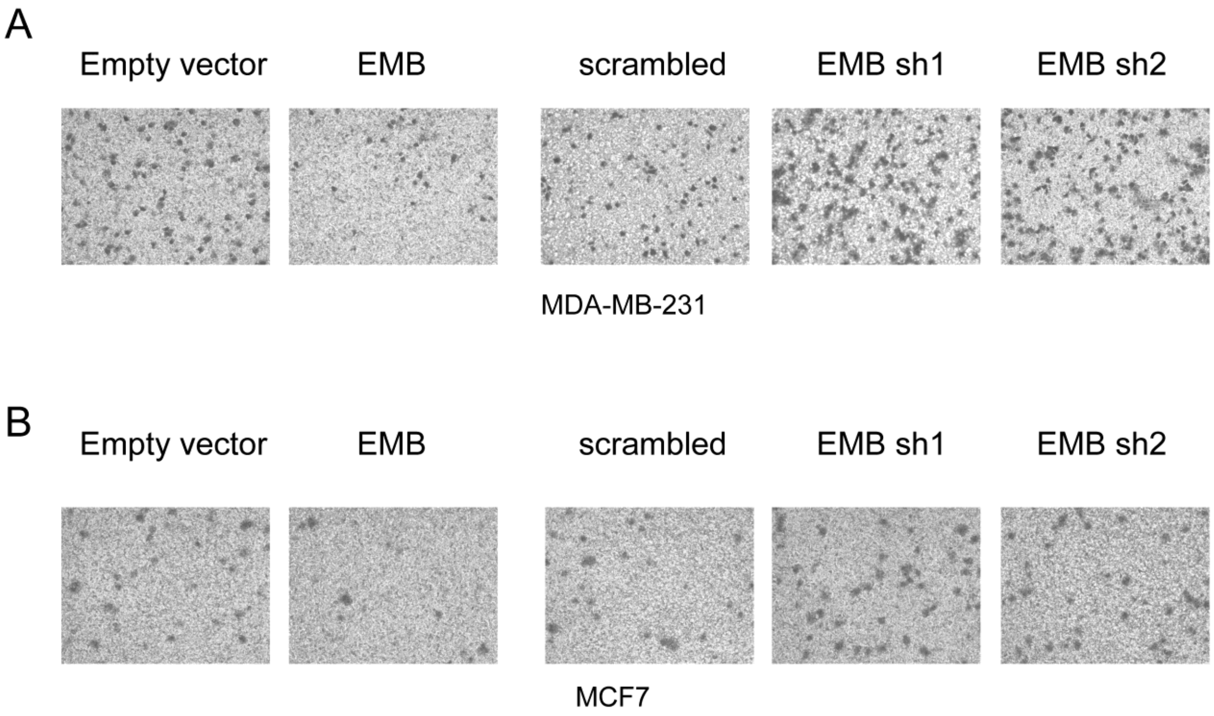

Figure S7: Migration assay performed in MDA-MB-231 and MCF7 cells. (A) MDA-MB-231 cells were lentivirally transduced with EMB expression vectors or shRNA vectors, and then transwell assays were performed and photographed. (B) MCF7 cells were lentivirally transduced with EMB expression vectors or shRNA vectors, and then transwell assays were performed and photographed.

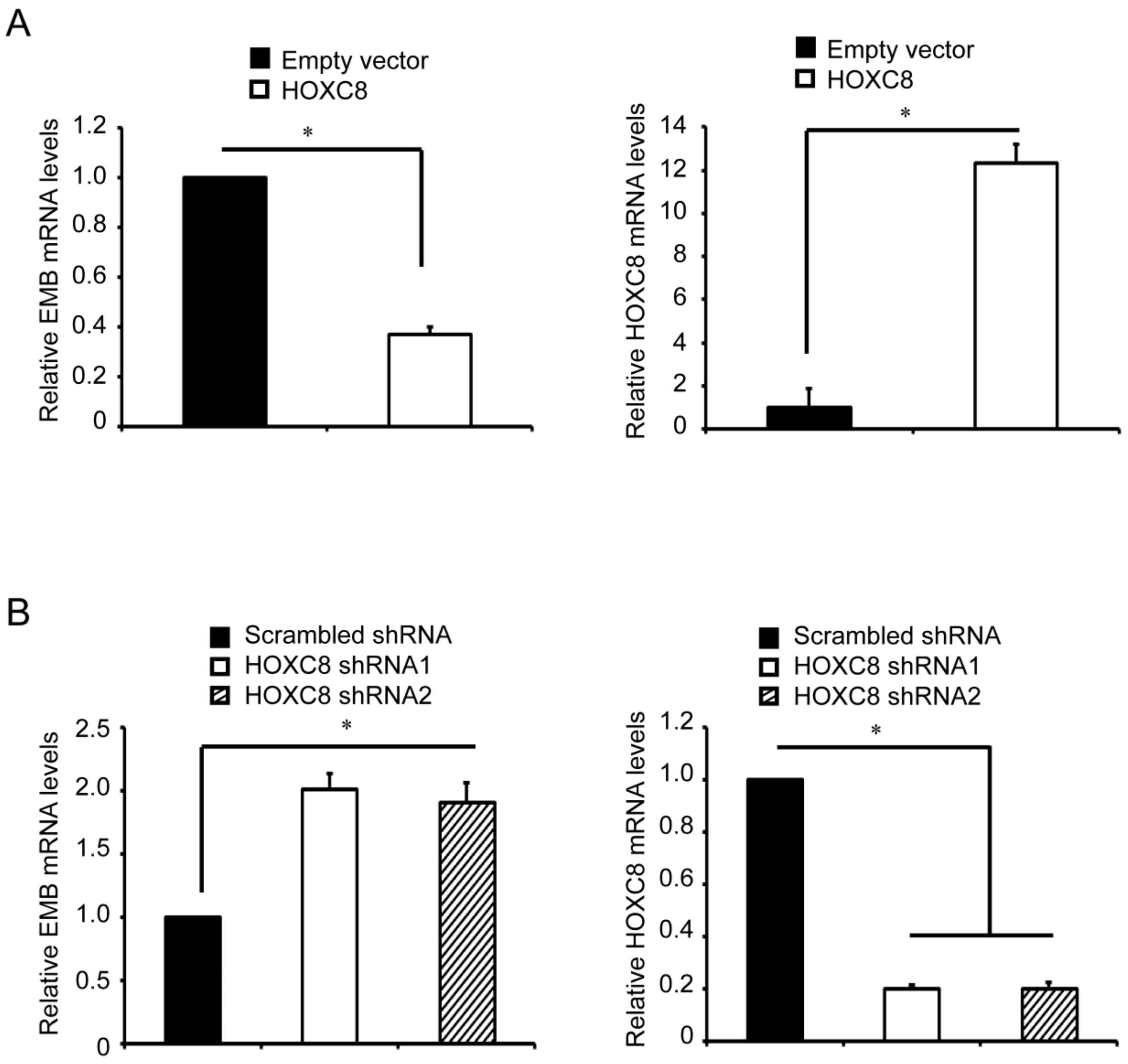

Figure S8: Effects of HOXC8 knockdown or ecto-expression on EMB mRNA levels in MCF10A cells. (A) MCF10A cells were lentivirally transduced with empty vector or vector encoding HOXC8, and total RNA was subjected to qRT-PCR to determine the level of EMB mRNA (left panel) and HOXC8 mRNA (right panel). β-actin and GAPDH mRNA were used as internal controls for standardization. Data are mean ± SE. n = 4. *P < 0.005. (B) MCF10A cells were lentivirally transduced with scrambled shRNA or HOXC8 shRNA, and total RNA was subjected to qRT-PCR to determine the level of EMB mRNA (left panel) and HOXC8 mRNA (right panel). β-actin and GAPDH mRNA were used as internal controls for standardization. Data are mean ± SE. n = 4. *P < 0.005.

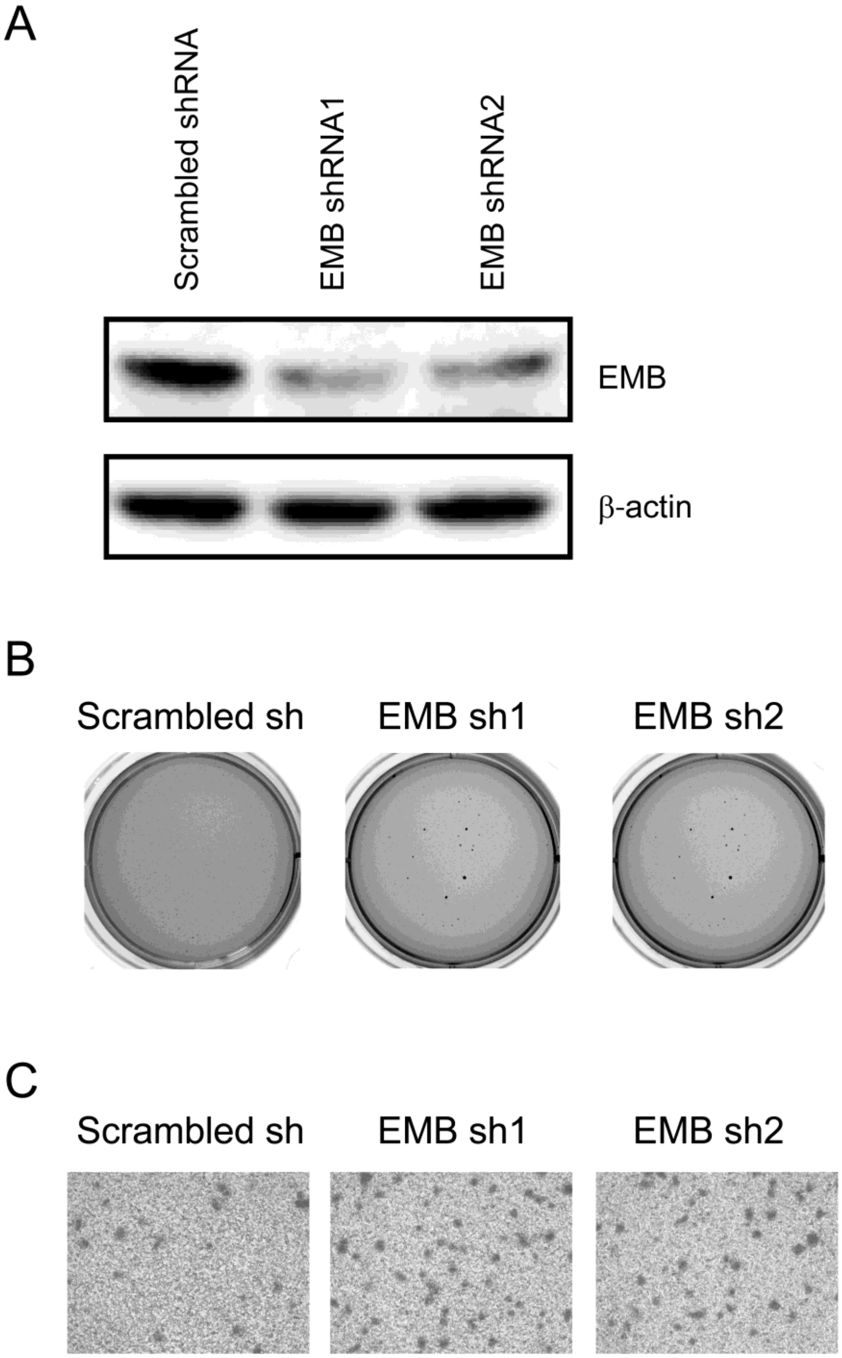

Figure S9: Embigin shRNA knockdown in MCF10A cells. MCF10A cells were lentivirally transduced with scrambled shRNA or EMB shRNA vectors. (A) Cell lysates were subjected SDS-PAGE and probed for embigin (EMB) and β-actin. (B) Soft agar assays were performed and photographed. (C) Transwell assays were performed and photographed.

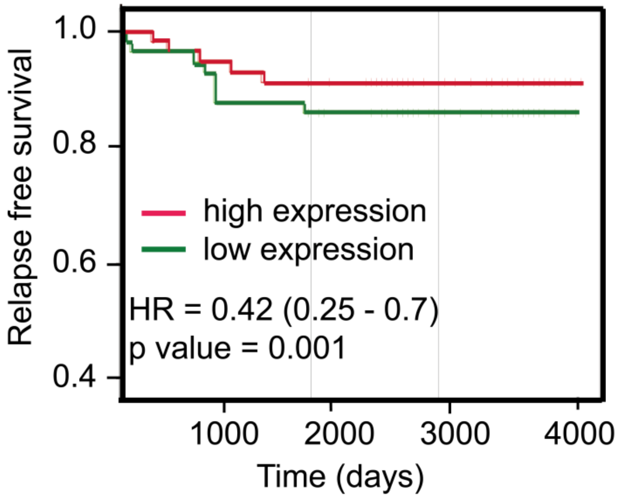

Figure S10: Breast cancer microarray data were analyzed using PROGgene web program with GSE19615. Kaplan-Meier curve showing recurrence-free survival of breast cancer patients with high or low embigin expression (logrank p = 0.001) using GSE19615 dataset. The total 114 patient samples were split into two groups according to median expression of embigin (high expression group: 57 samples; low expression group: 57 samples).

Table S1. Oligonucleotides used in real-time PCR, cloning and knockdown studies

**Primer Name**	**Sequence (5’ to 3’)**
Real-time PCR	
EMB forward:<br>EMB reverse:<br>HOXC8 forward:<br>HOXC8 reverse:<br>β -actin forward:<br>β -actin reverse:	TTTCCCCACCAGGCTAAAGT<br>TGGACCTGCACCCTAAAACA<br>GGCAAACTTACAGCCGGTAT<br>TTCAATCCGACGTTTTCGT<br>TGGATCAGCAAGCAGGAGTATG<br>GCATTTGCGGTGGACGAT
ChIP primers	
EBMChIPF1:<br>EBMChIPR1:<br>EBMChIPF2:<br>EBMChIPR2:<br>EBMChIPF3:<br>EBMChIPR3:<br>EBMChIPF4:<br>EBMChIPR4:	TTAACGCCGATATATTTTGAA<br>GGATGTTATAACGCATTAAAAGA<br>TCATATTATCTCACTGCCAA<br>GGTTAATTTGTAATTGGTACGTA<br>GTCAACAGTGTATAAGATTTAG<br>ATCTCAGAAAAGCCTCTTCT<br>TAAACATGACCCATTTCTC<br>AGGGACTCTTTTAAGGTTTT
Knockdown shRNA	
Scrambled shRNA<br>HOXC8 shRNA1<br>HOXC8 shRNA2<br>EMB shRNA1<br>EMB shRNA2	AAAACAACAAGATGAAGAGCACCAATTGGATCCAATTGGTGCTCTTCATCTTGTTG<br>AAAAGCAATATCCCGACTGTAAATCTTGGATCCAAGATTTACAGTCGGGATATTGC<br>AAAAGCCTCATGTTTCCATGGATGATTGGATCCAATCATCCATGGAAACATGAGGC<br>AAAAGAGTCATAACATATCACTGACTTGGATCCAAGTCAGTGATATGTTATGACTC<br>AAAAGCAGGTCCATATAGTCGTAATTTGGATCCAAATTACGACTATATGGACCTGC
EMB promoter cloning primers	
Forward:<br>Apa I reverse:	GCATTCTGGATTCATTGTAGAACTTTGATTC<br>AAGGGCCC TGGGAGGGGCCCGGCCGCCTTGCC

EMB expression vector	
EcoRI-Forward:	GAATTCTGGCGCCATGCGCGCCCTCC
BamHI-reverse:	GGATCCTCACTGGCCCAGAGACTCAT
